# Supplementary material for: Socioeconomic and demographic patterning of family uptake of a paediatric electronic patient portal innovation
Source: PLOS Digit Health. 2024 Oct 3;3(10):e0000496. doi: 10.1371/journal.pdig.0000496 (PMC11449342; doi:10.1371/journal.pdig.0000496)
Supplement: S1 Table — (DOCX) [file pdig.0000496.s001.docx]

S1 Table. Characteristics of included children by patient portal activation status.

| Characteristic | Portal activated  n=2682 | Portal not activated  n=1005 | Total (n=3687) |
| --- | --- | --- | --- |
| Sex - no. (%) |  |  |  |
| Female | 1302 (49) | 466 (46) | 1769 (48) |
| Male | 1380 (52) | 539 (53) | 1919 (52) |
| Missing | 0 | 0 | 0 |
| Age - no. (%)^a^ |  |  |  |
| Under 2 (<2yrs) | 231 (9) | 100 (10) | 331 (9) |
| Pre-school (2-4) | 437 (16) | 203 (20) | 640 (17) |
| Early childhood (5-10) | 1010 (38) | 413 (41) | 1423 (39) |
| Late childhood (11yrs and older) | 1004 (37) | 289 (29) | 1293 (35) |
| Missing | 0 | 0 | 0 |
| Ethnicity – no. (%)^b^ |  |  |  |
| White British | 1056 (40) | 308 (31) | 1364 (37) |
| Other White background | 320 (12) | 72 (7) | 392 (11) |
| Asian Bangladeshi | 102 (4) | 26 (3) | 128 (3) |
| Asian Indian | 120 (5) | 27 (3) | 147 (4) |
| Asian Pakistani | 176 (7) | 75 (7) | 251 (7) |
| Other Asian background | 136 (5) | 53 (6) | 189 (5) |
| Black African | 170 (6) | 77 (7) | 279 (7) |
| Other Black background | 124 (5) | 63 (6) | 177 (5) |
| Mixed / other ethnicity | 141 (5) | 50 (5) | 191 (5) |
| Prefer not to say / Not provided | 121 (5) | 109 (11) | 230 (6) |
| Primary language spoken – no. (%)^c^ |  |  |  |
| English | 2010 (78) | 621 (71) | 2631 (71) |
| Other language | 571 (21) | 257 (26) | 826 (23) |
| Prefer not to say / Not provided | 101 (4) | 127 (13) | 230 (6) |
| Family residence index of multiple deprivation (IMD) quintile – no. (%)^d^ |  |  |  |
|  |  |  |  |
| Most deprived (1^st^ quintile) | 426 (16) | 197 (20) | 623 (17) |
| 2^nd^ quintile | 765 (29) | 307 (32) | 1072 (29) |
| 3^rd^ quintile | 562 (21) | 206 (21) | 768 (21) |
| 4^th^ quintile | 477 (18) | 123 (13) | 600 (16) |
| Least deprived | 393 (15) | 133 (14) | 526 (14) |
| Unknown (primary residence outside the UK) | 59 (2) | 39 (4) | 98 (3) |
| Median number of associated non ophthalmic disorders or impairments (range ) | 4 (0 – 21) | 2 (0-15) | 3 (0 – 21) |

a. Pearson chi^2^ for difference in portal adoption across patient age categories =35.1, *p*<0.001

b. Pearson chi^2^ for difference in portal adoption across ethnicity categories =45.0, *p*<0.001

c. Pearson chi^2^ for difference in portal adoption across family primary spoken language =61.9, *p*<0.001

d. Pearson chi^2^ for difference in portal adoption across family residence deprivation index categories =21.9, *p*<0.001
