# Supplementary material for: Socioeconomic and demographic patterning of family uptake of a paediatric electronic patient portal innovation
Source: PLOS Digit Health. 2024 Oct 3;3(10):e0000496. doi: 10.1371/journal.pdig.0000496 (PMC11449342; doi:10.1371/journal.pdig.0000496)
Supplement: S2 Table — (DOCX) [file pdig.0000496.s002.docx]

**S2 Table.** **Primary spoken languages for families of children within study population**

| Language | Number of families |
| --- | --- |
| English | 2631 |
| Arabic | 142 |
| Urdu | 115 |
| Bengali | 76 |
| Somali | 59 |
| Turkish | 54 |
| Polish | 41 |
| Romanian | 36 |
| Panjabi | 25 |
| Portuguese | 24 |
| Tamil | 21 |
| French | 20 |
| Albanian | 16 |
| Hindi | 15 |
| Gujarati | 14 |
| Spanish | 14 |
| British Sign Language | 13 |
| Persian | 13 |
| Bulgarian | 11 |
| Greek | 9 |
| Lithuanian | 8 |
| Italian | 7 |
| Pushto | <7 |
| Chinese Mandarin | <7 |
| Kurdish | <7 |
| Primary spoken language for n≤3 families: Afrikaans; Akan; American Sign Language; Amharic; Bosnian; Braille; Cantonese; Czech; Dutch; German; Hausa; Hebrew; Hungarian; Igbo; Irish; Latvian; Lingala; Nepali; Romansh; Russian; Serbian; Slovak; Swahili; Telugu; Tigrinya; Twi; Ukrainian; Vietnamese; Wolof; Yiddish; Yoruba | |
