# Supplementary material for: Socioeconomic and demographic patterning of family uptake of a paediatric electronic patient portal innovation
Source: PLOS Digit Health. 2024 Oct 3;3(10):e0000496. doi: 10.1371/journal.pdig.0000496 (PMC11449342; doi:10.1371/journal.pdig.0000496)
Supplement: Attachment — Submitted filename: ReviewerComments_R!.docx [file pdig.0000496.s003.docx]

UCL GOS ICH, July 2024

We thank you for the opportunity to resubmit our manuscript.

We thank the reviewers for their helpful suggestions. Incorporation of the suggested changes has lengthened the manuscript to 3751 words.

A point by point response to reviewers follows:

1. Introduction

The introduction states there are inequalities already within childhood blindness. Could these existing inequalities affect the ability to examine these relationships within the population included in this study? This could be included in the discussion section – perhaps in relation to a larger discussion about generalisability.

- *We understand that the reviewer is asking whether socioeconomic patterning within the general population at risk resulted in selection bias with regards to the study population, and we thank them for the opportunity to expand on this important consideration.*

*The study hospital is a national centre of excellence, managing children who have been referred for specialised care by paediatric ophthalmologists elsewhere (in secondary and tertiary care units across the country). We have made this clearer in the manuscript {lines 352-353, 297} to support our statement that the study population within the paper is representative of those children in a high income setting with blindness or who need care for complex, blinding eye and vision disorders.*

*However, as the reviewer suggests, this study population may have resulted in an over-, or under-representation of those children from more vulnerable and underserved groups. We suggest that there has not been under-representation of those groups: this study population has an over-representation of those from ethnic minority backgrounds versus the general UK population (footnote added to table 1, line 114-115), and those living in deprivation, reflecting the “existing inequalities” highlighted by the reviewer (and as we now state in the discussion). Conversely our study population may hold an over-representation of these vulnerable groups. This would not negate our findings of differential engagement. Indeed, as we say in the discussion, a diverse patient cohort has been achieved in this study, with a resultant representation of those groups who are often under-represented in work on electronic health applications {line 303-304}.*

2. It might be helpful to include information about the incidence/prevalence of childhood blindness in the UK in the introduction for further context.

- *We have now included the incidence of childhood blindness {“Childhood visual impairment has an annual incidence of 1 per 1000,15 with 72% of children having other non-ophthalmic disorders or impairments.“ line 95-96}. We have also continued to make it clear that the study population is not limited to those children will have established visual loss. It includes those children who have been referred to specialised care services in order to prevent or avoid a blinding outcome {described in introduction as “…among those children affected with, or at risk of, visual impairment” lines 95}. Whilst the incidence of childhood blindness is known, the incidence of all blinding childhood disorders is not known.*

3. Materials and methods

The study design was described as cross-sectional, but based on the details in the methods I wondered if it might be a cohort study? Could this be clarified?

- *As we have not created the study sample with reference to the exposures of interest (in this study, the exposures are the sociodemographic characteristics) our conclusion is that this is not a retrospective cohort study.*

4. When examining the attendance rate at appointments over the past year, I was interested in knowing more about the distribution of the number of booked appointments per year in the patient population. Would it be possible a patient may not have a booked appointment and therefore non-attendance is not relevant? Was this variable explored as a possible predictor of engagement with the patient portal as it may indicate greater clinical need?

- *The reviewer makes an important point about ongoing need. We have clarified that the definition of “ongoing care” {line 382} included booked appointments, “(i.e., those who had scheduled outpatient consultations)”*

5. If this is not possible/relevant, then I wondered about severity of disease. There was some information provided on the types of eye conditions, but as a non-clinician I wasn’t certain about the severity/prognosis. Is it possible to group these conditions into relevant severity groups and consider that as a possible confounder and/or effect modifier in the analysis? Clinical need was discussed in the discussion section, but I think this could be further expanded, and perhaps sensitivity analyses around some of these other variables that could indicate clinical need could be explored.

- We thank the reviewer for suggesting this amendment. We have now made it clearer (see above) that the population of patients with childhood onset visual disability actually typifies rather than just represents the intersection of complex needs and multi-morbidity. We also describe that “Non-ocular disorders or impairments had been diagnosed in 2883 children (78%) in addition to their eye or vision disorder, with the number of additional disorders ranging from 1 to 21 (median 2).”{line 120-122}

We have undertaken a re-analysis using the number of additional impairments as a marker of additional need (“The number of additional non-ophthalmic diagnoses or impairments was also quantified as a potential proxy marker of clinical need.” {line 402-404}) which has not changed key conclusions but does allow us to report that the key associations are robust to adjustment for this marker of clinical need.

6. I know families that had not received care in the 6 months prior to the portal launch were excluded, but is there any information on how long the patients have been registered/attending the specialist centre and might that possibly be a predictor of portal use?

- *We do not have this marker but suggest that the key characteristics of interest are ongoing need and therefore ongoing drivers for use of the portal.*

7. I also wondered about inpatient vs outpatient attendance, were these types of appointments treated the same?

- *We have clarified above that the reference point for ‘ongoing care’ was ongoing outpatient care with regards to the sample population*

8. Many of the variables were ‘binarized’, but could the rationale for this be described further? Could collapsed categories have been considered (e.g. for ethnicity, using a version of the 5+1 census categories?) This was referred to in the discussion section on limitations but could be expanded.

- *We are grateful to the reviewer for allowing us to stress that whilst variables were binarized only where there was a supportive evidence base for this (e.g., children living in the lowest quintile for deprivation score, used consistently across medical literature). Ethnicity, as shown in tables 1-3, was treated as categorical data.*

9. Results

Table 1 – would be helpful if all variables included in the logistic models could be reported here (e.g. non-attendance to hospital appointments, etc). I also thought Table S1 was more informative than table 1 – the authors may wish to consider switching these, and perhaps including the chisq/p-values for the bivariate relationships.

- *These have now been included in table 1. We have not included the chisq/p-value for those relationships within the main text to avoid duplication of content in tables 2 and 3.*

10. Missing data was reported overall – but what about missing data by variable? Were there any variables in particular with a large proportion of missing data? Are the assumptions of complete-case analysis likely met and/or were any sensitivity analyses considered?

- *We have made it clearer that ‘Unknown’ in table 1 refers to ‘Missing’, so that readers can understand the low rate of missingness for all variables except the following:*

(i) *deprivation (unavailable for those children whose primary residence was outside the UK, as it is a UK based score) which is missing in 3% of cases)*
(ii) *ethnicity, where missingness is due to families not providing data, and has been treated as another category due to other literature suggesting that families do not withhold these data at random).*
11. Table 2 and 3 – it looks like some of the ethnicity categories are missing, could these be included?

- *We have excluded “Other” and “Mixed” categories as they are highly heterogenous groups. This is consistent with other approaches.*

12. Line 141 – might there be a typo in the 95% CI or the p-value for the adjusted OR for the ‘Black Other’ ethnicity category?

- *These tables have been amended following re-analysis.*

13. Discussion

The discussion covered many relevant factors to consider, but it would be helpful to get a broader sense of the literature and how this work might fit in to other studies about activation/uptake of patient portals.

- *We have added a paragraph within the discussion which expands on the points made in the introduction {lines 206-217}.*

14. Useful recommendations (e.g. incorporating non-digital interventions) were provided in the conclusions, but these could be discussed in more detail in the main discussion section.

- *We have ensured that the recommendations are made clearer within the discussion {line 238;243; 266;and 336,339} but have sought not to directly duplicate the key recommendations within the conclusion.*

15. Minor comments

- There is a period missing from the “author summary” paragraph, line 61.

*- This has been corrected.*

16. - Table 2, ‘Asian Indian’ has 3 SD recorded for the 95% CI, all others have 2 SD.

*- This has been corrected.*

Point by point response to Reviewer #2:

17. Foremost, the study is framed by an introduction and the discussion section focused on a basic exposition to the influence of health information technology at the international level, including barriers to access in low resource countries. This discussion is interesting and reasonably well referenced, but unrelated to the study itself. Given the study focus on patient portal use and personal health records access among children and their families, the introduction should focus on that topic, presenting concisely, what is known and where the gaps are that this particular study will address. The current introduction does not clearly indicate the motivation for the presented study.

- *We have removed this section from the introduction. However, we have kept the statement “Across the globe, in diverse health care settings, socioeconomic and demographic factors such as income level and ethnicity have a marked influence on access to health services and subsequent health outcomes” within the discussion as we feel that this is a key consideration for those who design and implement portal services.*

18. The manuscript presents a clear justification for the use of a population of children under the age of 18 seen in an optimal logic clinic, however, the larger manuscript repeatedly refers to the population as involving children with complex medical conditions. This is confusing to the reader. While this reviewer respects the idea that Complex eye disease has value as a burden on patients and their families, most readers are likely to imagine the concept of complex medical conditions in children referring to illnesses such as cancer, hematologic diseases, or diabetes that involves multiple organs. The manuscript should be precise about its focus on eye disease from the outset and throughout.

- *We have reviewed our manuscript and suggest that we have been precise, whilst also taking this chance to use this patient population as an ‘exemplifier’ of complex health disorders.*

19. With that said, and in this reviewer's experience, complex multi organ chronic disease among children tends to be a stronger motivator for patient portal use than single organ disease.

- *Our new findings on multimorbidity (e.g., inclusion as a variable) now address this point. Please see response (5) above. We also now note in the discussion that our findings can be used to evidence the intuitive assumption of a positive association between multi-morbidity and portal use, as earlier reports were not able to find this independent association {lines 205-217}.*

20. The authors should also ensure that their literature review captures the specific state of knowledge as it relates to pediatric patient portal use among children with chronic eye disease, which currently is not presented in the introduction section.

- *We have now stated that “There is a lack of evidence on the patterns of adoption for patient portals and similar health technologies within this patient population.” {line 103}*

21. The manuscript presents findings from different childhood age groups but does not justify how the age groups are combined. Further, there is no information about children aged 16 to 18 despite the methods indicating that children with this age group will be included in the study.

*- We thank the reviewer for pointing out an error – 16-18 year olds are included within the categorisation of 11 years and older (i.e., secondary school education age in the UK) within this paper. We have now ensured that the manuscript tables are consistent. The justification (i.e., use of school age thresholds) is now made explicit {line 381-2}. We have made it clear that paediatric care in the UK covers children up to their 18^th^ birthday {line 367}. We have also recognised the importance of understanding the proportion of the cohort who are aged 16 -18 years old {line 118}.*

22. The manuscript indicates that they developed a home grown patient portal called MyGOSH. The manuscript does not describe the portal, or indicate why they chose a homegrown portal – which may limit generalizability – over the use of the integrated my chart portal that is available for their epic EHR system.

- *We thank the reviewer for suggesting we make it clearer that MyGOSH is “a child and UK centred adaptation of the MyChart application”* {lines 357-358}

Reviewer #3:

23. The sequencing of the sections may need to fit in better with the PLOSOne format.

From line 292-376 Material and methods, Study design, Inclusion/exclusion criteria, Data collection, Statistical analyses should be before the Results and Discussion section.

- *We have followed the guidance on manuscript structure as found here:* [*https://journals.plos.org/digitalhealth/s/submission-guidelines#loc-manuscript-organization*](https://journals.plos.org/digitalhealth/s/submission-guidelines#loc-manuscript-organization)

*We are happy to follow any amendments suggested by the editorial team.*

Minor considerations

24. Referencing

In general, the paper may be improved with more appropriate references:

• Line 227-228 – how was non-engagement deduced as a ‘soft marker’?

- *We have now referenced the statement that “A ‘soft’ marker of non-engagement may be reluctance to self-provide family ethnic background” with this citation: (*[*https://pubmed.ncbi.nlm.nih.gov/37217292/*](https://pubmed.ncbi.nlm.nih.gov/37217292/)*)*

25. • Line 260-261 – are there any peer-reviewed metrics that the authors could consider utilising to understand the perceived need for using the portal? If so please provide the reference. If not, could authors consider qualitative or consumer designed approaches.

- *We have now used multi-morbidity to try to quantify understand perceived need: (duplicated from point 5 for ease):* We have now made it clearer (see above) that the population of patients with childhood onset visual disability actually typifies rather than just represents the intersection of complex needs and multi-morbidity. We also describe that “Non-ocular disorders or impairments had been diagnosed in 2883 children (78%) in addition to their eye or vision disorder, with the number of additional disorders ranging from 1 to 21 (median 2).”{line 120-122}

We have undertaken a re-analysis using the number of additional impairments as a marker of additional need (“The number of additional non-ophthalmic diagnoses or impairments was also quantified as a potential proxy marker of clinical need.” {line 404-406}) which has not changed key conclusions but does allow us to report that the key associations are robust to adjustment for this marker of clinical need.

26. • The authors may benefit from including some reference around the need for consumer engagement in co-designing digital health intervention and portals.

- We have added this citation of the very good paper suggested by the reviewer (although note that this review was limited in scope around co-development, with more of a focus on PPI in research)
