# Supplementary material for: Socioeconomic and demographic patterning of family uptake of a paediatric electronic patient portal innovation
Source: PLOS Digit Health. 2024 Oct 3;3(10):e0000496. doi: 10.1371/journal.pdig.0000496 (PMC11449342; doi:10.1371/journal.pdig.0000496)
Supplement: Attachment — Submitted filename: R2 comments.docx [file pdig.0000496.s004.docx]

We thank both the reviewers for their earlier comments which have helped, as they noted, make the manuscript stronger.

In response to Reviewer 2’s comments “The only remaining item the authors should consider is that it remains confusing that there is an apparent focus on the single chronic medical condition of blindness, while the study itself appears to include all comers (i.e., not just patients with blindness). This is confusing to the reader.”

We have made it clearer that childhood blindness (rather like child mortality) is a health outcome rather than simply a single medical condition:

*“Childhood visual impairment (VI), which is a health outcome as well as a medical condition, has an annual incidence of 1 per 1000.^15^ VI in childhood is a strong marker of broader child health, with close correlation with child mortality, and with 72% of children with VI also having other non-ophthalmic disorders or impairments.^15,16^ It also shares with broader child health a strong patterning with socioeconomic family status” (lines 88-92)*

Oher comments:

Journal Requirements:

Please review your reference list to ensure that it is complete and correct.

- This has been done

Preflight Analysis and Conversion Engine (PACE) digital diagnostic tool

- We confirm that no image problems were identified through the PACE uploading. We confirm that we have reviewed the preview of our figure rendered on a simulated PLOS journal page for image clarity and content.
